# Dispensing care?: The dosette box and the status of low‐fi technologies within older people’s end‐of‐life caregiving practices

**DOI:** 10.1111/1467-9566.13455

**Published:** 2022-03-10

**Authors:** Tessa Morgan, Robbie Duschinsky, Stephen Barclay

**Affiliations:** ^1^ School of Nursing University of Auckland Auckland New Zealand; ^2^ Department of Public Health and Primary Care University of Cambridge Cambridge UK

**Keywords:** actor–network theory, ageing, elderly care, family/kinship, health technology/ technology assessment, narrative method

## Abstract

Technology has been lauded as a solution to range of challenges presented by ageing population internationally. While the lion‐share of scholarship has focussed on high‐fi, digital technologies, there has been a recent shift to exploring the contributions mundane, low‐fi technologies make to older people's daily lives and our understandings of health, illness and care more broadly. Drawing from serial narrative interview data collected with 19 married couples aged 70 and over living in the U.K., this article explores the way one medical technology—the dosette box—was taken‐up and deployed in their end‐of‐life caring process. Informed by actor–network theory and critical feminist scholarship, this article considers how the dosette box played an active role in structuring relationships, scheduling daily care activities and enforcing medical compliance. In doing so, we suggest that the dosette box provided an unexpected companion and ‘weapon of the weak’ for older partner's attempting to assert their expertise and power while caring. We also explore how the dosette box demanded an even higher level of regular, vital care from older partner's once introduced into the home, thus entrenching the physical and emotional demands of dispensing care.

## INTRODUCTION

### Techno‐optimism and older people's health

Technology has been lauded as a solution to range of challenges presented by ageing population internationally (Biehl & Moran‐Thomas, 2009; World Health Organisation., 2017; Xiong, 2021). Technologies have been celebrated for enabling older people to remain independent and at home for as long as possible, by promoting their mobility, security, (Zhang et al., 2020) cognitively stimulation, social connectivity(Chopik, 2016) and self‐care practices (Lattanzio et al., 2014). Such technologies range from elaborate monitoring systems in ‘smart homes’ to wearable wristbands to keep older people safe, through to robotic companion pets to combat loneliness (Cowan & Turner‐Smith, 1999; Pols & Moser, 2009; Sixsmith & Gutman, 2013). Technological innovations have been welcomed as ways to promote healthy ageing understood as ‘process of developing and maintaining the functional ability that enables wellbeing in older age’(Sixsmith & Gutman, 2013; United Nations., 2020). Technologies are not by definition digital, as ‘technology’ can relate to any machine, device, artefact or object that are used in ‘a manner of accomplishing a task especially using technical processes, methods or knowledge’ (Merriam‐Webster, 2021). Nevertheless, the lion's share of research and industry excitement centres on the promises of ‘intelligent’ assistive technologies and digitalised information and communication technologies to improve older people's lives (Buse et al., 2018). Indeed, in our digitised era older people's access to technology is being framed as a ‘human right’(Fang et al., 2021), a claim strengthened in the post‐COVID‐19 world where digital health care is being promoted as a fundamental systems change for delivering health care to older people (Monaghesh and Hajizadeh, 2020; Bar‐Tur et al., 2021).

### The dark side of technology?

The tenor of current scholarship is overwhelmingly orientated towards emphasising older people's ‘eagerness’ to learn new technologies (Vaportzis et al., 2017) and challenging ageist discourses that older people are unable to use technology (Joyce & Mamo, 2006). Nevertheless, some research has drawn attention to the darker side of technological innovation. First, studies with older people have addressed the negative emotional experiences, such as ‘techno‐stress’, emerging from frustrations of learning new technologies and societal expectations that digital literacy is required for societal inclusion (Nimrod, 2020). Second, studies have highlighted older people's concerns about technology such as privacy erosion from technologies like invasive smart home monitoring devises (Ienca et al., 2021; Mortenson et al., 2016). Other documented fears include concerns that technologised forms of care will supplant in‐person care consultations (Lindberg et al., 2021). In addition, Joyce and Mamo (2006) have made a powerful case that within biomedicine, the aged body has been increasingly constructed as a set of ‘age‐related diseases as well as a site for continual restoration and improvement’(pp. 99–100). Older people therefore face a moral imperative to use life‐extending technologies and deny the realities of ageing even at their end of life (Joyce & Mamo, 2006).

Other scholars have critiqued the new promises of technology for reinforcing pre‐existing power structures that further embed exploitation (Latimer, 2018; Schiller, 2019). Scholars are increasingly pointing to the exacerbation of the digital divide along the lines of intersecting, persistent inequalities such as age, ethnicity and class (Fang et al., 2021). Concerns have also been raised that through technologising care older people are conceived of as dependent and ‘at risk’ by virtue of needing monitoring and surveiling by robot devices (Wigg, 2010). Many high‐tech care solutions have also been critiqued for presenting band‐aids to wider structural problems such as the declining space for and importance afforded to social connection and care in modern societies (Fraser, 2017; Putnam, 2000; Schiller and McMahon, 2019). To this end,feminist scholars Hobart and Kneese (2020) contend that self‐care devices such as fit bits and smart phones enable individuals to ‘maintain productivity in the face of adversity and exhaustion’(p. 4). Yet in doing so they offer ‘a fresh iteration of the Weberian Protestant work ethic’ rather than something actually nurturing or emancipatory (Hobart and Kneese, 2020, p. 4). Mort et al. (2015) have attested to this dynamic in the context of older people's engagement with telecare, which appears to offer greater control and personalisation of care options. In practice, however, sick people are often left to provide more care for themselves despite not necessarily having the adequate training, equipment or physical ability to effectively do so.

### Mundane technologies and methodological opportunities

There remain important limitations in this blossoming field of research. Most prominently there are long‐standing concerns that studies tend to take a deterministic view of technologies, placing a far greater focus on the acceptability and up‐take of technology rather than the dynamic ways technologies are used by older people (Rodeschini, 2011). A connected concern is that older people are commonly assigned to the role of object rather than subject in the development of technology (Brittain et al., 2010; Ienca et al., 2021). In consequence, few studies put ‘older people's meaning making, creativity, and bodies at the centre of analysis of technology, science and health’(Joyce & Low, 2010, p. 172). Another concern is that while science and technology approaches are widely used in the sociology of health and illness, enquires have thus far privileged technological innovation over mundane care thus limiting our scope of knowledge about older people's daily uses of technology (Buse et al., 2018).

There has been an important shift, spear‐headed by special collections in *Sociology of Health and Illness*, to explore the contributions of mundane, low‐fi technologies to our understandings of health, illness and care (Buse et al., 2018; Joyce & Low, 2010). For example, Loe (2010) poignantly demonstrated how older women combine a bundle of technologies such as thermoses, warm clothes, walkers and telephones to enable their safe and comfortable movement around their neighbourhoods. Subsequent studies have highlighted how mundane technologies such as beds, buttons and doors actively shape and structure the meanings and identities produced through caring for older people (Buch, 2018; Buse & Twigg, 2018; Cleeve et al., 2020). In the context of end of life, Ellis and Muller (2020) has drawn attention to how food becomes a means for families to discuss and orientate their care as well as a way of temporalising the end‐of‐life trajectory. She observed that ‘food fights’ occurred where family members disagreed over the relative weighing of nutrients versus the discomfort of eating, in turn shaping their feelings about the food and each other (Ellis and Muller, 2020). This finding reflects the profound affective impacts of ordinary material objects and practices, something that is currently receiving wider sociological attention (Berlant, 2011; Latimer, 2018). As studies about ordinary technologies focus on the ongoing, emergent, processural and affective qualities of interacting with these technologies, such enquires have also re‐orientated definitions care from being a functional task or moral orientation to a set of practices and relations (Buse et al., 2018; Mol et al., 2010; Winance, 2010). What remains underexplored, however, is the way that older people themselves engage with technologies to care for others and their self (Cleeve et al., 2020).

### The dosette box

This article contributes to the growing body of scholarship about older people's use of technologies by considering older partner's engagement with the dosette box. The dosette box is a Swedish‐manufactured pill box which has NHS endorsement (NHS England., 2020) and is designed to ensure that people remember to take their medication at the correct time and in the prescribed dosage (Dosett, 2015). It is offered as a tool for family members and professional carers supporting an older person who require daily medications (Helping Hands., 2020). Aesthetically, its form follows its function: it consists of a clear plastic tray that organises medicines into separate compartments for different times of the day for each day of the week. Each box contains a week's worth of medication and has clear labels with times and days (Helping Hands., 2020). While some research to date has been conducted about this particular technology, most has been situated within clinician‐orientated journals and focussed on whether families are receiving the appropriate amount of support to administer such medication (Kripalani et al., 2007; Kwan et al., 2013; Thomas et al., 2018). Evidence confirms that older spouses are less likely to receive support when administering medication, underscoring calls to improve their training and support (Joyce et al., 2014). In these discussions, the dosette box features as a device for improving medication adherence, though its efficacy is unclear (Gillespie et al., 2015). Through characterising medication provision as a linear process that one can succeed or fail at, less consideration has been made of the role the dosette plays in wider end‐of‐life care practices.

### Older spousal end‐of‐life caregiving

The present study also addresses another neglected feature of older people's care practices: older spousal carers. While older carers emerged as a category in the 1990s (Wenger, 1990), it is only very recently that they been considered key actors in the informal care force in economically developed nations (Henwood et al., 2017; NHS England., 2019). In the UK, the Social Market Foundation estimates that there are currently two million carers aged 65 and over, 417,000 of whom are aged 80 and over (2018). Carers UK reported that the total number of carers has risen by approximately 11% since 2001 with the number of older carers increasing threefold (35%) (Carers UK., 2015). Concerns have been raised by the charity sector that older people have been ‘left to fill the gap’ of inadequate care provision of the social care system (Age UK., 2017). Little remains known about their care practices, though some recent studies have explored the health impacts of caring and the identity‐work involved in everyday caring (Morgan et al., 2020). Few studies have explicitly addressed the material aspects of the caring practices of older spousal carers, and less still about their use of technologies to provide end‐of‐life care (Morgan et al., 2020). The aim of this article is therefore to understand how the dosette box mediates older spousal carer's experiences of providing daily care for their partners approaching their end of life.

## METHODOLOGY

### Theoretical framework: ANT and neglected things

This inquiry is broadly shaped by the claim of actor–network theory (ANT) that ‘all entities in the world are constituted and reconstituted in shifting and hybrid webs of discursive and material relations’(Blok et al., 2020, p. xx). In this view, objects, as well as subjects, are capable of being actors shaping the conditions of possibility of everyday life and mediating human action through forming patterned networks of heterogenous materials (Law, 2002b, Maller, 2015), hence the interconnected neologism actor‐network (Latour, 2005). This perspective is deepened by contributions of feminist scholars in material culture and Science and Technology studies who argue for the academic relevance of ‘quiet, routine, almost unnoticed’ low‐fi technologies to understanding the contexts and relations through which care is ‘felt and lived’ (Buse & Twigg, 2018; Maller, 2015; Pink et al., 2014). Recent enquires have shown how attending to neglected things helps to unpack a more dynamic, sensitive vision of caring practices by attending to the temporal, spatial, affective and processural aspects of care (Buse et al., 2018). In addition, the act of developing a ‘speculative commitment to neglected things’ furthers our understandings of asymmetrical power relations operating through technological assemblages by asking questions like ‘why is this neglected?’ and ‘who (human or non‐human) is expected to create, maintain, or cover‐up such devalued practices’? (Puig De La Bellacasa, 2011). By sensitively responding to these questions about knowledge and practice, Puig de la Bellascasa (2011) contends that we can begin to care for those people and things involved in devalued labours as well as opening alternative ways of structuring our actor‐networks.

ANT is well‐suited to this task as it comprises an intellectual practice concerned with the introduction and incorporation of technologies by pursuing deep, descriptive case studies that attend to interplays of human and non‐human actors (Latour, 2005; Mol, 2010). An ANT approach ‘follows’ research participants’ interests and interactions in order to comprehend their actor‐networks (Blok et al., 2020; Latour, 2005). For this study, the dosette box made itself a focus by emerging as an animated participant in the interview process. Participants rattled the box and opened its seals and frequently described their use of the box as well as its contents, to orientate and talk about their caring. Even when direct attention was not drawn to it, the box's mere presence on surfaces such as the living room or dining room tables in all participants homes pointed to the important role it played in the chronic care infrastructure of participants’ daily regimes (Langstrup, 2013).

While pillboxes have existed since Ancient Greece, (Martin, 2006) the modern pillbox has been contingent on a number of technological developments including the invention of gelatine pill casings 1830s (Morris, 2019) and development of synthetic plastics at the turn of the 20th century (Bijker, 2012). Such technologies have become increasingly widely used with the rise of chronic illness, as people live far longer due to the assistance of medical technology (Buch, 2018). The widespread need for such medical management technologies is supported by a 2014 NHS survey that found almost that almost half of all UK adults are currently taking daily prescription medication, with two million pensioners taking seven different prescription drugs per day (NHS England., 2014). This growing requirement for individuals to be responsible for dispensing their own daily medicines, rather than trained health‐care professionals, accords with wider shifts in health‐care models from formal institutions to the community (De Nooijer, 2020; Foucault, 2006, 2008; Heaton, 1999). Foucauldian scholars have located such changes to the ongoing diffusion of the medical gaze whereby the family since the 1980s has become increasingly implicated in monitoring the bodies of their family members and their selves while medical practitioners have growing ability to surveil their efforts and their homes (Rose, 1990).

### Data collection

This study is shaped by a narrative approach that treats storytelling as a fundamental means through which people make sense of the world, themselves and others (Kleinman, 1988; Mishler, 1984). Narrative approaches focus on the content, form and context of individual cases (Wiles et al., 2005) and endeavour to explore ‘the contradictions of social interaction and self‐presentation’ in such accounts (Bury, 2001, p. 278). Narrative inquiry may focus in the first instance on talk, but this can and should be carefully situated with reference to the material contexts which shape, organise and at times interrupt discourse (Thomas, 2010).

Fieldwork took place between August 2018 and August 2019 with participants living at home with their partner in Cambridgeshire or West London, United Kingdom. Participants took part in up to three semi‐structured in‐person audio‐recorded narrative interviews held approximately a month apart. The longitudinal approach enabled the one interviewer (TM) to build rapport with participants while capturing their unfolding priorities and storylines, which enhanced the detail and depth of the data (Murray et al., 2009). To qualify for the study, participants had to be 70 or over and looking after their partner at home who had a diagnosed palliative condition. A horizontal sampling method was used, utilising strong and weak ties as ‘bridges’ into new social networks: participants were recruited via two General Practitioner (GP) surgeries, two former carers and the dissemination of a recruitment flyer to carers’ organisations (Geddes et al., 2017). All potential participants were first contacted via the telephone to explain the study, to confirm their willingness to take part and arrange in‐person meetings. All participants provided written consent at the beginning of each interview. Two spousal carer participants agreed to interviews but subsequently withdrew from the study, one because he was himself diagnosed with terminal cancer and another because her husband only had days to live.

In total, 41 interviews were conducted with 20 participants across 17 couples. On average, interviews lasted one and a half hours but ranged from 30 min to 6 h (Table 1). Reasons given for not taking part in subsequent interviews were generally high care demands and/or physical and mental decline of one or both partners. Participants were offered the choice to be interviewed either together or on their own (Rose and Bruce, 1995; Wadham et al., 2016). Due to the high level of cognitive‐impairment amongst end‐of‐life partners, only two couples both became participants. Twelve severely cognitively impaired partners were present during the interviews. Field notes were also taken which enabled the researcher to capture dynamics between couples, along with observations about the material aspects of care.

**TABLE 1:** Characteristics of participants

Couple number	Partner providing care	Age	Ethnicity	Care‐receiving partner	Age	Ethnicity	Care recipient diagnosis	Number of interviews
1	Wife	78	Scottish	Husband	78	Scottish	Alzheimer's	3
2	Wife	74	White British	Husband	74	White British	Alzheimer's	1 (Caregiver died)
3	Husband	84	White British	Wife ^*^	78	White British	Advanced Frailty	3
4	Wife	70	White British	Husband	85	White British	Vascular Dementia (deceased)	3
5	Wife	73	White British	Husband	85	White British	Front temporal Dementia	3
6	Wife	77	Welsh	Husband	89	Jamaican	Cancer and Alzheimer's	2 (Couple evicted)
7	Wife	80	White British	Husband	84	Irish	Cancer, Vascular Dementia, Stroke	3
8	Husband	75	White British	Wife ^*^	73	White British	COPD	3
9	Wife	78	White British	Husband	82	White British	Vascular Dementia, COPD	1
10	Wife	71	White British	Husband	72	White British	Alzheimer's and Vascular Dementia	3
11	Wife	73	White British	Husband	72	White British	Parkinson's	3
12	Husband	85	White British	Wife	85	White British	Alzheimer's	2
13	Wife	73	White British	Husband	79	White British	Parkinson's/Lewy Bodies Dementia	2
14	Wife	80	Indian	Husband	84	Indian	Alzheimer's	2
15	Wife	77	White British	Husband	82	White British	Vascular Dementia	3
16	Wife	89	Italian	Husband	89	Indian	Lewy Bodies Dementia	2
17	Husband	80	White British	Wife	87	White British	Stroke	1
		M=77.5		^*=^care recipient also participated	M= 81.05			41

*= Care recipient also participated.

### Data analysis

Analysis began during the interview process with TM recording field notes directly after each interview (Green et al., 2007). Each audio file was sent for transcription immediately so that TM could read each transcript and make notes about key stories ahead of each follow‐up interview. TM discussed these observations with participants to support the transparency and trustworthiness of the findings (Lincoln and Guba, 1985). Once each case had been analysed, patterns of meaning were identified across cases (Riessman, 2008). Below we present three narrative case studies that exemplify the complex roles the dosette box played in shaping and animating older couples daily end‐of‐life care practices. The centrality of the dosette box in daily care routines and the relational nature of using it to take medication were qualities shared across the data set. However, the three case studies were selected because these participants provided the most sustained attention to the dosette box.

### Radhika: ‘I have also these problems with me’

Radhika used her dosette box to craft her and her husband Rahul's daily care rituals around optimising health. As a response to the initial interview question ‘what's it like to look after your spouse?’, she slowly rose from her seat on the couch to retrieve the two dosette boxes which sat one atop another on a white bookshelf in the centre of her small council apartment. Delicately opening one morning tab with her gnarled, arthritic fingers, Radhika meticulously explained the symptoms (like memory) or organ (like kidney) to which each of the eight pills in her husband's dosette box related. She notably lingered on the large oval vitamin D tablet which she had acquired from a health store and subsequently added to both of their boxes to ‘improve their mood’ (int 1). She then repeated this process with her own dosette box.

Following a description of what was in the box Radhika then turned to describing how it operated as a central organiser of their day; attesting to the ways that ‘technologies help to shape ways of living with disease’(Pols & Moser, 2009, p. 161). The dosette box timetabled the day in tandem with other objects such as the small whiteboard which Radhika referred to as ‘his chart’ propped up on the dining room table. The day was divided into hour time slots from 5 am to 8 pm, with medication time appearing thrice on the white board. The temporality inscribed by the dosette box not only mediated Radhika's actions but also her sense of self. This was most clearly outlined in her response to a question about whether she saw herself as a carer:

Definitely mam because I have to start from the going to bed and have to finish. I give him medicine and eye drops and then ask him to do this thing then tell him to go to bed. And in the morning again his medication and all these things. So yes I am his carer, his full day carer. [laughs] (int 1)

With the box in hand, Radhika asserted the importance of her own self‐care as fundamental to being able to sustain Rahul's care. Her dosette box worked as her accomplice allowing her to follow doctor's orders. She also linked her medical regime to her wider aspirations of keeping active, acknowledging health discourses endorsing the personal responsibility of active ageing (Stenner, 2011).

Cause I was wondering what are the other ways you care?

[Radhika taps the dosette box with her finger]

… oh yeah I've seen that before. So those are your ones?

This is my vitamin D and one medicine every Monday I take for my arthritis. Once a week this is the tablet.

Do you find that your arthritis makes it hard to care for him?

That is what I'm saying. Because the doctors say ’keep yourself active’ and that's all. So nothing. But if I get more pain then I apply [raises gel bottle]. I can't fold my fingers also. It used to be like that. Twice or thrice I had an injection. That's why I am keeping myself active doing exercise if I like it or not. And not only once, I have divided into four times. Immediately before getting up I do some exercises lying down only.

It sounds like you are really taking doctors’ orders.

Yes I have taken them as part of my life [laughs](int 1)

While committed to the principle and its authority, Radhika found it increasingly difficult in practice to remain active due to her arthritis and declining hearing and Rahul's declining memory and worsening asthma meant that they were both currently housebound. What made the situation frustrating was Rahul's reluctance to ‘enrol’ in Radhika's interpretation of their dual boxes as mirroring their mutual need (Callon, 1984). Rather she felt that her husband claimed the ‘patient’ role in the household, thus positioning his needs above hers. This was exhibited during the second interview where a return to discussing the dosette box sparked Radhika to challenge her husband (sitting on the couch adjacent to us) about his failure to recognise her health problems:

I was wondering when you said the husband has to look after the wife?

I don't, that's what I told him because he always say [mimics husband] ‘I am old I am sick like that’.

What do you say to that?

That's what I'm thinking what do you give him in reverse this thing so he should realise I said, ‘I am also old I have also these things problems with me’. (int2)

Recognising the emotional and physical impact her daily caring was having on her, particularly when compared to her expected cultural norms that ‘in India the ladies don't take so much of the burden which I have got’, Radhika was compelled to seek formal support to sustain care at home. With the help of a care navigator she arranged a health‐care assistant (HCA) for one hour a day to assist her with Rahul's daily personal cares. The dosette box featured as an actant in this care hand‐over process, no less because it was an object through which Radhika could clearly teach and judge the quality and timeliness of her new health‐care assistant's care (Andersen and Bengtsson, 2019). The interaction of the HCA and the dosette box resulted in Radhika's caring role shifting from doing the care to instructing and overseeing it:

He's good? He's up to your standard?

So now he knows how many tablets have to be given what time … and now he does the bed then making breakfast and then doing the utensils which are tea and breakfast utensils. And so one hour passes like that his medication and…

You do you find that helps you?

It helps me. Get in because he does everything what type of things I want I always get at him ‘this is not the way to do this’ so he has become perfect now huh. (int 2)

Ultimately, the dosette box was an important feature of Radhika and Rahul's caregiving arrangement as it temporally structured their daily care practices and helped Radhika's cultivate a sense of self‐efficacy in providing care. However, the dosette box required Radhika to provide complex, continuous monitoring that fuelled feelings that her ‘problems’ were secondary to her husband's.

### Helen: ‘I press the button’

For Helen, the dosette box gave the semblance of control and empowerment in an otherwise tumultuous caregiving narrative that had spanned over twenty‐five years in which crisis had become ordinary (Berlant, 2011). Despite ‘hav[ing] to be attentive all the time’ to her husband Barry's care needs resulting from his end‐stage Vascular Dementia, Chronic Obstructive Pulmonary Disease (COPD) and Heart Failure, Helen found it difficult to talk about the subject. In an interview otherwise full of strained silences which were filled by her mid‐life daughter who also participated in the interview, it was the dosette box that finally got Helen talking:

I: Are you in charge of medication?

Helen: I do, yes. After he came out of hospital there were heart pills, new ones, and I was going, [argh] I can't cope. There was so many to arrange and I was frightened I wouldn't get the right ones. I just happened to go in the chemist one day and I said ‘do you ever do things like a dosette box that I can have a monthly box’, and they did. It's made a tremendous difference.

Daughter: They're amazing.

Helen: I put them out for him. I press the button and press the thing out for him, but at least I haven't got to count out 12 or 15 tablets every morning, again at lunchtime and in the evening (int 1).

By framing herself as the person who had the idea to get one in the first place, as well as being the person who would subsequently ‘press the button’ Helen presented herself as a ‘knowing subject’ enacting her agency in a ‘transformative and transcendent sense’ to improve her situation (Berlant, 2011, p.136). ANT thinkers posit that human actors always navigate their agency in relation to technology as well as other non‐human actants rather than independently from them (Law, 2002a). Armed with this insight, it is interesting to consider the ways that Helen's agency was actually mediated by the dosette box.

The box was required in the first place to resolve the problem of how Helen could continue to manage Barry's complex medical regime despite his unwillingness to adhere to doctor's orders. His obstinance had left her feeling that she could ‘run, scream, cry’ from their caring arrangement (int 1). Incorporating the dosette box into their home ushered in an immediate relief as it instantly became her ally by containing, locking and hiding medication from Barry's reach and helping to share the load of policing his dangerous attempts at self‐care:

Helen: Yes, but he did take the wrong ones. That's why I hide them now because he did once take them when I wasn't at home because he wanted to go to bed to get to sleep. He'd obviously got very anxious. Now I hide them. I don't want that to happen. He takes headache pills. I've stopped that now. A day I put out if he wants eight because he was taking the full ten that I would put out because of his headaches. (int 1)

The materiality affordances of the dosette, including the process of counting, placing, checking, administering, resulted in Helen developing a form of ‘tactical knowledge’ which she used to cultivate her feelings of control (Pink et al., 2014). This was supported by the habitual acts required by the dosette box which helped Helen to cultivated an infinal ‘holding environment’ within the home keeping him alive, and thus enabling her sense of successfully performing her wifely duties (Berlant, 2011, p. 146). The dosette box thus enabled her to dispense care even when both Helen and Barry's feelings about each other soured. This was illustrated best when Barry claimed that Helen was taking over his life through requiring his medical compliance, which in turn made her feel like he did not recognise or appreciate the emotional and physical labours involved in keeping him alive. As Helen shared:

Helen: Today he said again ‘you're taking over my life. I'm able to decide what I want you're trying to rule me’. I said ‘I'm not. I'm trying to prevent you from having pneumonia again. That's why you've got the thickener in your drinks. It's a silent killer they told me, you know so therefore’. (int 1)

The authority and knowledge gained through using the dosette box gave Helen the means and courage to surveil her husband's General Practitioner (G.P.). Helen felt they were ‘at the end of the line’ with their current G.P. because he had done little recently to review medications and vary the contents of the dosette box. Helen felt the G.P. was thus not as committed as he ought to be to bettering her husband's health or at least improving his behaviour to make caring for his care less ‘obviously demanding [laughs]’ (int 1). While caring left Helen ‘shattered’, with assistance from the dosette box she was able to perceive herself as fulfilling normative expectations to care for her spouse, while attaining some sense of control over her constant state of crisis.

### Joan: ‘There's a slip between cup and lip’

Joan referred to herself as a ‘very can‐do woman’ caring for her second husband for the last nine years as he had developed Parkinson's and end‐stage Lewy body Dementia. Having just returned home from hospital herself after having collapsed due to exhaustion, Joan surprisingly orientated her second interview around a range of issues involving her husband Richard's medication:

**I:So it's about the right medication?**

Joan: Yep I'm lucky I've got a very good chemist up the road. But there's lots of little what shall I say we've got an old English saying ‘there’s often a slip between cup and lip’ which is just an old one. But my G.P. had phoned me and said she needed to have a talk to me and I must have it must have been when the painters were here and I missed the call and they didn’t bother to call back. And this time when I went up to get his dosette boxes the chemist said to me ‘Joan’ he said ‘I’m a little bit worried because they’ve moved two of the tablets from his morning one and a night time one’ and I said ‘what are they’ and he told me and I said ‘well I don’t understand’ and he said ‘this is the prescription that he was working from and it just said Rivastigmine cancelled’. (int 2)

Here, the box was an actant in the care chain connecting herself with the actions and thoughts of her chemist, G.P. and the supplier of Richard's dosette box. As the passage above attests, all actants in the network took up‐keep. The dosette box needed checking for errors, the pharmacist requires sweet‐talking, Joan and GP had to be readily available at the end of the phone.

Joan emphasises that the reason for these fragile relations were a product of the wider health‐care system which epitomised through the old British idiom ‘*there's often a slip between cup and lip’*. While the NHS was ostensibly committed to principles of universal health care, Joan highlighted that the layers of bureaucracy and lack of integration of the system result in poor care in practice. As such the dosette box was a body‐guard against health‐care professionals’ potential mishaps:

So you can imagine if I hadn’t had a… because when you see that like that [shakes dosette box] you know I only count the number I know there’s two in there two in there five in there five in there but there must be people who are living on their own.

**Yeah who aren’t maybe…**

Who just open it or the carer opens it and just takes the tablets you know, as you say if you’re not on the ball really all the time it can be very hit and miss. (int 2)

The importance of always being ‘on the ball’ attests to the vital stakes involved in acquiring ‘tactical knowledge’ (Pink et al., 2014). Such knowledge was challenged when her husband's care entered the hospital. Framing herself in the ‘educated consensus’, Joan continued that it was widely known that being in hospital was dangerous for people with Parkinson's, a piece of knowledge shored up by her experiences of hospital doctors throwing away Richard's dosette box upon entering hospital:

**Do** **you find when he is in hospital do you have to give him some of his tablets?**

They won’t let me, they won’t even… it’s very strange. This is what I get now which saves me hours of time. [Rattles dosette box.] Because he’s on as you can see a lot of tablets but when he goes into hospital I take this with me but they won’t let him use them because they’re not in their sealed packets. (int 2)

Joan interpreted this as a sign of displacement of her position in the care hierarchy; shifting from ‘conductor to second fiddle’ as Lowson et al. (2013) has conceptualised it. She found this particularly affronting because at home the same box signalled her place at the top of the care hierarchy above her HCA who she monitored in a similar way to Radhika, and at least on par with her local pharmacist. This attests to the ways that technologies always contain multiple agendas depending on the arrangement of the network and context of their deployment (Latimer, 2018). For Joan, this enshrined the importance of keeping care at home which with the support of her dosette box she felt this was something she very much could do.

## DISCUSSION

This article provides a novel contribution to the literature about older people's engagement with technologies. This article supports growing calls to attend to mundane technologies alongside more hi‐fi equivalents, as a way to holistically understand older people's daily lives, and more broadly capture the intricacies of providing end‐of‐life care (Buse et al., 2018). This article offers a complex account of older people's agency with regard to technologies, challenging more deterministic accounts of older people's use of technology (Rodeschini, 2011). For example, by drawing from an ANT perspective, we demonstrate how the dosette box played an active role in structuring relationships, scheduling daily care activities and enforcing medical compliance. This supports sociological insights into the way very ordinary objects can play important roles in life‐building and life‐making practices even if their contributions are not immediately obvious (Berlant, 2011; Puig De La Bellacasa, 2011).

This account of the dosette box as an active participant in end‐of‐life care presents a far more enlivened and nuanced account of medical management than what is currently depicted in clinician‐orientated scholarship to date (Joyce et al., 2014). This analysis supports Ellis's calls for further research centring on material‐based practices of older people, particularly and in the context of end of life (Ellis and Muller, 2020). This provides an alternative to current scholarship that often privileges questions of care on a discursive level (Morgan et al., 2020),and tends to focusses on material practices enacted on older people without considering their ongoing and affective engagement with such technologies (Cleeve et al., 2020). Given the shift to provide older people's support and care at home and the growing responsibilities for families to provide medications, such insights into low‐fi technologies and care are timely.

This analysis builds on insights about the role technologies play in cultivating particular kinds of knowledge through ongoing caring practices. We contend that the dosette box, as a teacher, fostered a form of ‘tactical knowledge’ (Pink et al., 2014) as it demanded pressing, placing, counting and monitoring, which older carers could then use to cultivate their sense of self‐efficacy and expertise for their partner's care. Drawing attention to this function also attests to just how much relational work was required of partners to successfully engage with the dosette box which is typically conceptualised as a ‘self‐care’ technology, a point echoes similar insights made about blood pressure pumps (Weiner & Will, 2018). By making visible the knowledge gained through engagement with the dosette box, this analysis builds on calls in scholarship and policy to recognise the currently neglected expertise of family carers (Morgan et al., 2020). Sociologically, this article deepens accounts of the circulation of power between hospital and home (Foucault, 2008; Heaton, 1999; Langstrup, 2013). We contend that the dosette box, owing to its clear plasticity, made the operations of medical power partially transparent as participants could see what medications were being prescribed in what doses and make their own judgements about the appropriateness of prescribing. The dosette box thus provided opportunities for older carers to reverse the ‘medical gaze’ back onto the medical establishment (Biehl & Moran‐Thomas, 2009; Heaton, 1999). This was exhibited where older carers collaborated with the dosette box to surveil and make assessments about the medical management of doctors, health‐care assistants and pharmacists involved in their partner's care. The dosette box also interestingly functioned as a barometer of medical professional's commitment to providing high quality care; information which was then leveraged in decisions about whether to persevere with particular health‐care professionals.

In these ways, we suggest the dosette box could be considered a ‘weapon of the weak’ as John Scott (1985) terms it, as it enabled a relatively powerless group to improve their everyday lot through very ordinary practices of resistance. This insight builds on the body of social theory exploring the ways patients and their relatives have sought to subvert pre‐established avenues of power in order to make medical professions see and listen to them so as to improve their situation (Farge & Foucault, 2012; Petryna, 2002). These findings specifically deepen Langstrup's idea of ‘chronic care infrastructures’ as networking users into the clinics, by suggesting the dosette box made clinics accountable to the domestic also (Langstrup, 2013). Notably, this recalibration of the carer‐health‐care professional power dynamic did not translate when older patients entered the hospital system, indicating that the dosette box's power was also heavily contingent on the authority afforded by the domestic context (Lowson et al., 2013). This analysis therefore points to ways that technology has the potential to improve older people's lives when it contributes to reciprocal relationships between health‐care professionals, patients and family. This analysis attests to the importance of relationships, not the technology itself as the central ingredient for good care, a lesson worth reiterating in light of the increasing push for digitalised solutions to older people's care (Monaghesh and Hajizadeh, 2020; World Health Organisation., 2017).

Importantly, this analysis also contributes to the growing pool of scholarship addressing the ambivalent position of technology in daily life, including in older people's care (Joyce & Mamo, 2006; Mort et al., 2015). While improving these women's handle on their responsibilities and their husbands, the dosette box demanded an even higher level of regular, vital care from participants. This aligns the dosette box with the ambivalent status of technologies such as fit bits, smart phones and telecare which at once promise control over oneself but result in doing more health‐orientated work in the service of the wider biopolitical regime (Hobart and Kneese, 2020; Mort et al., 2015). Precisely because the dosette box is introduced in all the cases above to improve circumstances and promote on‐goingness, we suggest it ought to be regarded as a ‘cruelly optimistic’ technology. Berlant (2011) offers this concept cruel optimism as a way of understanding processes like providing the care that centre around an aspiration, in this case, sustaining one's partner's life, yet in the process of pursuing them, wear out the aspirants. This was attested to in the way that the examples where older wives were frequently worn out, physically and mentally, in the process of fulfilling the requirements and promises of the dosette box.

Another way that this technology could be interpreted as cruelly optimistic was through the hierarchies produced through materially mediated care practices. This was most clearly illustrated when participants’ needs were subordinated to the needs of their partners or medical professionals despite appeals to the dosette box for their authority and legitimacy (Buch, 2018). This finding builds on Latimer's (2018) observation that material processes can create ‘thresholds’ through which people must pass through to become legitimate patients, with such imaginaries associated with cultural scripts around appropriate need. That two older partners from the same couple could use the same medical technology with dramatically different results attests to how technologies are always intricately linked with other social and physical processes, in these cases, historically imbalanced patriarchal family compositions and communication issues associated with cognitive decline. Such findings complement recent scholarship emphasising the affects produced through engagement with technologies and material objects and how these can in turn shape, for better or worse, dynamics of family relationships at the end of life (Ellis and Muller, 2020; Winance, 2010). By caring for the dosette box, this article usefully problematises notions of harmonious family dynamics so frequently romanticised at the end of life (Broom et al., 2016). While technology has a lot to offer to improving older people's daily lives, we contend that there remains darker sides of incorporation which must always also be considered.

### Strengths and Limitations

The strength of this study is its longitudinal nature and open‐ended narrative structure which enabled older participants to express what they felt was meaningful about their caregiving experience and the dosette box to become visible through the interview and analysis process. A limitation of this study is that it only comprises heterosexual couples. Future research could usefully focus on older LGBTQ+ couple's caregiving at end of life as existing evidence suggests that this group tends to privilege friends and other non‐kin actors in their care networks, which could possibility add another relay of power surrounding the dosette box (Hughes & Cartwright, 2014). Due to their impaired cognition, participants’ husbands were not explicitly included as participants in this study, thus limiting our ability to understand their views and experiences of the dosette box. Future research ought to use tools such as process consent to ensure that they included as participants as it would help further understandings around how dementing bodies engage with technologies (Schillmeier, 2019). A final limitation is that this study reports interviews about conversations with health‐care professionals rather than direct observations of those conversations, something future research could usefully explore. This study was conducted in the U.K. which has a relatively comprehensive health and social care welfare system. Future research could consider the function of dosette boxes and other medical management technologies in health‐care systems that are more privatised and consequently place more emphasis on the health consumer's purchasing power (provided they have the income to support this).

## CONCLUSION

This article captures the ambivalent nature of technology in older people's daily lives, thus countering the dominant narrative of technology as the exalted solution for internationally ageing population. This article demonstrates how older partners engaged with the dosette box to make their daily caring more manageable and meaningful; yet in turn often result in people shouldering more caring responsibilities required by the pill organiser. By exploring older partners affective and processural engagement with the dosette box, this article also contributes to the growing body of sociological scholarship centring mundane low‐fi technologies as important access points to understanding daily care practices and the wider power structures in which care is dispensed.

## AUTHOR CONTRIBUTION

Tessa Morgan: Conceptualization (equal); Data curation (equal); Formal analysis (equal); Funding acquisition (lead); Investigation (lead); Methodology (equal); Project administration (lead); Supervision (supporting); Validation (equal); Writing – original draft (lead); Writing – review & editing (supporting).

## Data Availability

The data that support the findings of this study are available on request from the corresponding author. The data are not publicly available due to privacy or ethical restrictions.
